# The epidemiology of postnatal depression in Ethiopia: a systematic review and meta-analysis

**DOI:** 10.1186/s12978-020-01035-1

**Published:** 2020-11-19

**Authors:** Bereket Duko, Dereje Wolde, Yonas Alemayehu

**Affiliations:** 1 Faculty of Heath Sciences, College of Medicine and Health Sciences, Hawassa University, Hawassa, Ethiopia; 2 School of Public Health, Curtin University, Perth, Australia; 3 Department of Medical Laboratory, Sodo Christian General Hospital, Wolaita Sodo, Ethiopia

**Keywords:** Postnatal depression, Epidemiology, Associated factors, Ethiopia

## Abstract

**Background:**

Postnatal depression is among the common mental health problems that occur during the postnatal period. However, it is left undiagnosed in low- and middle-income countries including Ethiopia. Therefore, this systematic review and meta-analysis aimed to systematically summarize the available evidence on the epidemiology of postnatal depression in Ethiopia and suggest recommendations for future clinical practice.

**Methods:**

The Preferred Reporting Items for Systematic Reviews and Meta-Analyses (PRISMA) guideline was followed to conduct this systematic review and meta-analysis. We searched PubMed, SCOPUS, EMBASE and Google Scholar databases for the relevant articles that assessed the prevalence of postnatal depression in Ethiopia. We used a random-effect model to conduct a meta-analysis. We conducted a subgroup and sensitivity analysis to explore the source of heterogeneity. Cochrane Q- and the I^2^-test were used to check the heterogeneity of the included studies. The presence of publication bias was also checked by visual inspection of symmetry and Egger's test.

**Results:**

The pooled estimated prevalence of postnatal depression in Ethiopia was 20.1% (95% CI 12.7–30.2). The pooled prevalence of postnatal depression in the studies that were conducted in community settings and used the Patient Health Questionnaire to assess postnatal depression [16.6% (95% CI 8.90–28.99)] was lower than the prevalence in studies based in institutions and that used the Edinburgh Postnatal Depression Scale [23.2% (95% CI 14.50–28.5)]. Further, in a leave-one-out sensitivity analysis the prevalence of postnatal depression ranges between 15.4% and 25.4%. Unplanned pregnancy [AOR = 3.46, 95% CI (2.37–5.04)], age between 15–24 years [AOR = 1.72, 95% CI (1.11–2.68)], marital problems [AOR = 3.07, 95% CI (2.36–3.99)], experiencing the death of infant [AOR = 3.41, 95% CI (1.91–6.09)] and history of substance use [AOR = 3.47, 95% CI (2.17–5.56)] were associated with the increased odds of postnatal depression in Ethiopia.

**Conclusion:**

The prevalence of postnatal depression in Ethiopia was high. Therefore, the concerned body should give due attention to improve reproductive health services through early detection of risk factors of postnatal depression.

## Background

The postpartum period is a challenging phase characterized by significant biological, physical, social change, and also with an increased risk of postnatal depression (PND) [1]. PND can be explained by the presence of various groups of depressive symptoms and syndromes which prevail within the first 12 months of childbirth [2, 3]. Numerous signs and symptoms such as loss of interest in daily activities, loss of appetite, disturbed sleep, decreased concentration, and negative attitude toward new baby, suicidal ideation, guilty feeling, and easily fatigability define the presence of PND [3, 4].

Reports from the World Health Organization (WHO) in 2017 suggested that more than 322 million people were suffered from depression [5] and out of this, approximately 29.9 million people were from Africa. The Global Burden of Diseases study in 2015 systematically analyzed the data of 17 low-and middle-income countries (LMICs) and reported an 18.4% prevalence of PND [6]. Previous systematic review and meta-analysis conducted in 2012 to assess the pooled prevalence of perinatal common mental disorders including depression in LMICs reported 19.8% [7]. Further, studies conducted in African countries such as South Africa and Ethiopia reported that the prevalence of postnatal depression ranged between 30 and 50%, suggesting one in every ten women in the postnatal period experiences depression [8–11].

A significant number of women are at higher risk of developing depression in the postnatal period due to different risk factors. The most common risk factors has been reported in the previous studies include domestic violence [12], younger age [13, 14], low-income [15], lack of social support [16], unplanned pregnancy [15–18], anxiety during pregnancy, stressful recent life events, low education, past history of mental illness including depression and history of abortion [19–22].

Women who suffer from postnatal depression may have problems with daily functioning, and may also be at increased risk for suicidal thoughts and behaviors [6]. This can also result in poor mother to infant relationships, infectious illness to infants, and affect children’s growth and development [23]. Besides, it could result in emotional, behavioral, and cognitive problems in the later life of the child [23, 24].

Although postnatal depression is treatable and clinical as well as behavioral interventions can be delivered by specially-trained non-specialists, there is limited evidence about the prevalence and related risk factors for postnatal depression in Ethiopia. Complementing this, a study conducted in 2016 revealed a lack of evidence on the prevalence of pre-and postnatal mental health problems in low-and-middle-income countries, suggesting the urgency of pooled estimated data on the topic to inform policymakers to address the disease burden [25]. Therefore, this systematic review and meta-analysis aimed to systematically summarize the available evidence on the epidemiology of postnatal depression in Ethiopia.

## Methods

### Research design

The Preferred Reporting Items for Systematic Reviews and Meta-Analyses (PRISMA) guideline was followed to conduct this systematic review and meta-analysis [26]. The review was conducted using a predesigned protocol for database searching, data extraction, inclusion–exclusion criteria, quality evaluation, data synthesis, and analysis (Additional file 1).

### Search strategy and selection process

We searched PubMed, SCOPUS, and EMBASE databases for the possible relevant articles that assessed the prevalence of postnatal depression in Ethiopia using the following terms and keywords: (epidemiology OR prevalence OR magnitude) AND (perinatal depression OR postnatal depression OR maternal depression OR postpartum depression) AND Ethiopia. Furthermore, we repeated searching using the following search terms and keywords: (perinatal depression OR postnatal depression OR maternal depression OR postpartum depression) AND (associated factors OR correlates OR risk factors OR determinants) AND Ethiopia. We also searched EMBASE and SCOPUS using database-specific subject headings. Besides, we scanned the reference lists of the included literature.

### Eligibility criteria

Our review included the observational studies that assessed the prevalence and associated factors of postnatal depression in Ethiopia. We have included all the studies irrespective of the publication date. Commentaries, editorials, reviews, and letters to editors were excluded from the review.

### Methods for data extraction and quality assessment

The review was guided by the Preferred Reporting Items for Systematic Review and Meta-Analysis (PRISMA) [27]. The data extraction was performed by two reviewers (BD and MT) independently. We utilized a predefined data extraction form to extract data. The authors' name, year of publication, the sample size, study design, study setting, and the instrument used to assess postnatal depression, as well as associated factors along with adjusted odds ratios, were extracted from each study. The Newcastle–Ottawa Scale (NOS) which is adapted for cross-sectional studies was used to check the quality of studies included in the review [28]. The tool used to assess postnatal depression, the quality of the methods, sample size, sample representativeness, and comparability between participants were the domains NOS scale to check the quality of studies included in the review. The agreement between the evaluators was appraised using agreement beyond chance (unweighted kappa) (BD and DW). The levels of poor, slight, fair, moderate, substantial, and very high levels of agreement were depicted by the values 0, 01–0.02, 0.021–0.04, 0.041–0.06, 0.061–0.08, and 0.081–1.00, respectively [29].

### Data synthesis and analysis

We conducted a meta-analysis using the Comprehensive Meta-Analysis software version 3.0. The random-effect model was used to pool the overall prevalence of postnatal depression in Ethiopia [30]. The Cochrane Q-and the I^2^-statistics were used to assess the heterogeneity between the studies [31]. The values of 25, 50, and 75% represented a low, medium, and high level of heterogeneity respectively [32]. We have also conducted the subgroup and sensitivity analysis to assess the possible source of heterogeneity. Publication bias was evaluated by using Egger's test and visual inspection of the symmetry in funnel plots. The level of significance was set at P < 0.05.

## Results

### Identification of studies

**Fig. 1:**
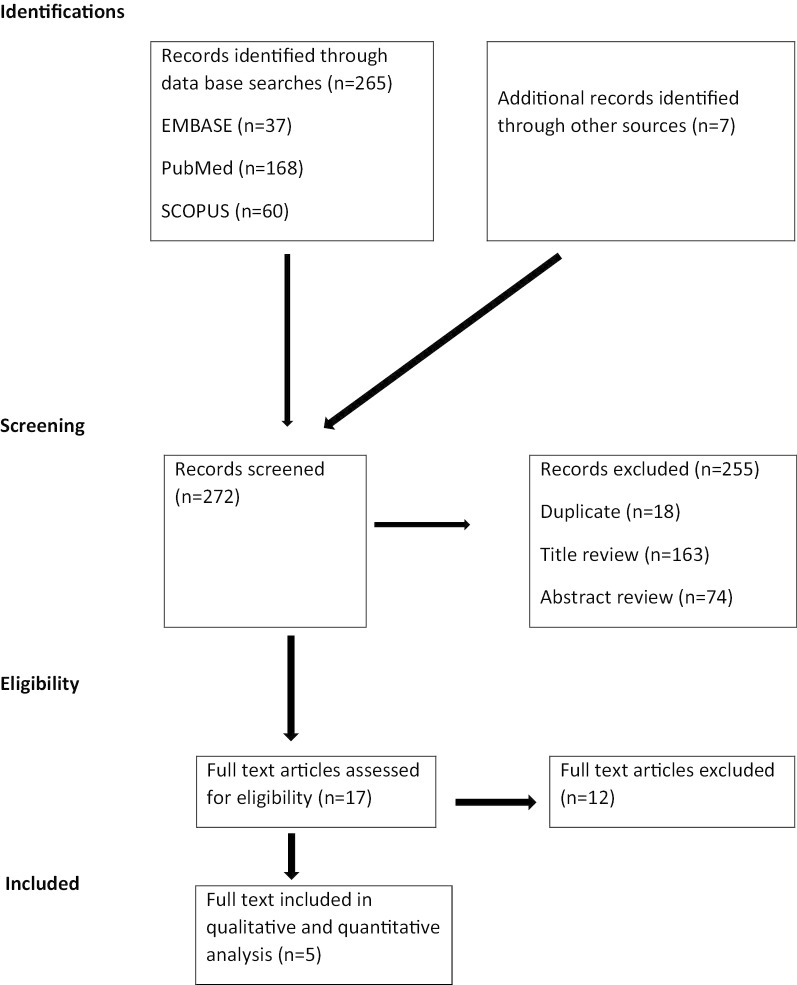
PRISMA flowchart of review search

A total of 272 articles was identified both on an electronic database and manual search. We excluded 255 publications during the review of titles, duplicates, and abstracts as they did not fulfill the eligibility criteria (Fig. 1). Therefore, 17 full-text articles were included for further screening and 12 of these were excluded. Finally, five full-text articles were included in the systematic review and meta-analysis.

### Characteristics of included studies

Table 1 depicted the characteristics of the included studies. A total of 4751 study participants were included in the current review. The studies included in the review were conducted between 2014 and 2017. All studies included in the current systematic review and meta-analysis used a cross-sectional study design. Out of these, three studies were institution-based cross-sectional whereas two studies were community-based studies. Three studies were conducted in South Nation Nationalities and the People Regional States (SNNPRS), one was conducted in the Amhara region and one was conducted in Addis Ababa. Three studies used the Edinburgh Postnatal Depression Scale (EPDS) and two studies used the Patient Health Questionnaire (PHQ9) to assess postnatal depression.

**Table 1:** Characteristics of included studies: epidemiology of postnatal depression in Ethiopia

Author name, year	Region	Sample size	Study design	Settings	Data collection tool	Prevalence (%)	NOS
Kereie et al. [11]	SNNPRS	408	Cross-sectional	Institution-based	EPDS	33.82	7
Fantahun et al. [18]	Addis Ababa	618	Cross-sectional	Institution-based	EPDS	23.30	9
Toru et al. [14]	SNNPRS	456	Cross-sectional	Community-based	PHQ-9	22.40	8
Shewangizaw et al. [19]	Amhara	122	Cross-sectional	Institution-based	EPDS	13.11	7
Azale et al. [20]	SNNPRS	3147	Cross-sectional	Community-based	PHQ-9	12.2	9

*PHQ-9* Patient Health Questionnaire, *EPDS* Edinburgh Postnatal Depression Scale

### The quality of the included studies

The Newcastle–Ottawa Scale (NOS) which is adapted for cross-sectional studies was used to check the quality of studies included in the review. The quality evaluation of the included studies based on the NOS revealed that three studies were of high methodologic quality whereas two studies were of moderate quality. The agreed levels between the authors regarding the quality of the studies included the meta-analysis ranged from moderate to very high levels of agreement (Table 1).

### A meta-analysis of the prevalence of postnatal depression in Ethiopia

**Fig. 2:**
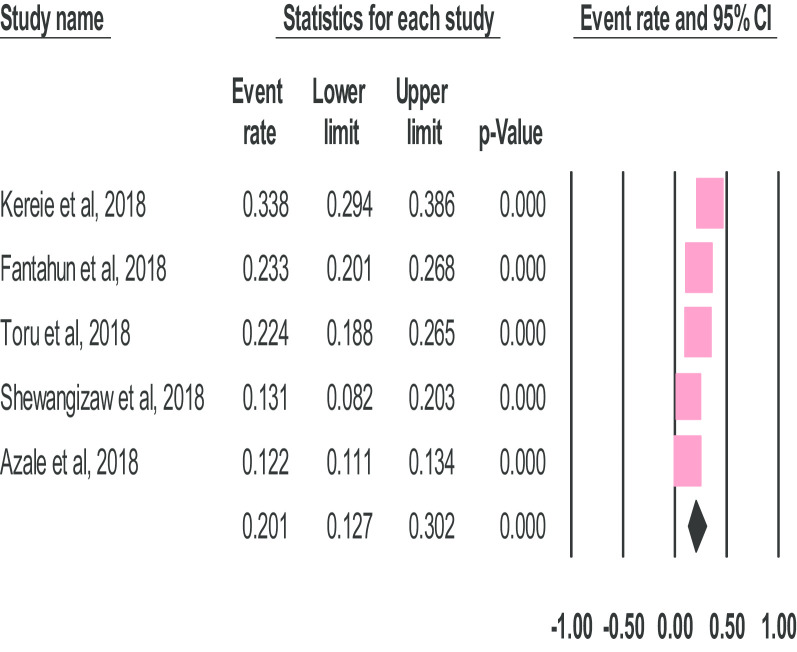
The prevalence of postnatal depression in Ethiopia: a meta-analysis

The overall pooled prevalence estimate of postnatal depression in Ethiopia was 20.1% (95% CI 12.7–30.2) and the observed heterogeneity was significant (*I*^2^ = 99.408%; Q = 154.300, df = 4, p < 0.001) (Fig. 2).

### Subgroup and sensitivity analysis

**Fig. 3:**
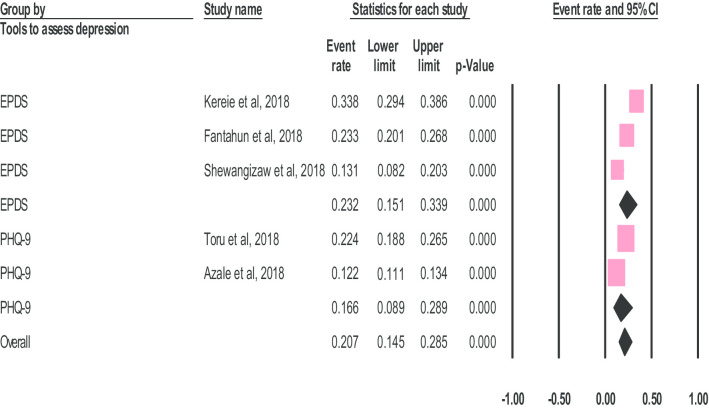
The prevalence of postnatal depression using depression assessment tool: a meta-analysis

We performed subgroup and sensitivity analysis to identify the source of heterogeneity among the studies. The pooled prevalence of postnatal depression in the studies that were conducted in community settings and used the Patient Health Questionnaire to assess postnatal depression [16.6% (95% CI 8.90–28.99)] was lower than the prevalence in studies based in institutions and that used the Edinburgh Postnatal Depression Scale [23.2% (95%CI 14.50–28.5)] (Fig. 3). The heterogeneity was significant in both studies, that were conducted in the community and institution-level (*I*^2^ = 97.085; Q = 34.303, df = 1, p < 0.001) and (*I*^2^ = 91.971; Q = 24.909, df = 2, p < 0.001) respectively.

Furthermore, we conducted a leave-one-out sensitivity analysis to identify the possible source of heterogeneity in the pooled meta-analysis of the prevalence of postnatal depression in Ethiopia. This analysis showed that the overall prevalence was strong and not dependent on a single study. The pooled prevalence of postnatal depression ranges between 15.4% (14.3–16.5%) and 25.4% (23.3–27.6%) (Table 2).

**Table 2:** Sensitivity analysis of prevalence of postnatal depression for each study being removed at a time: prevalence and 95% confidence

Study excluded	Prevalence	95% CI
Kereie et al. [11]	15.4	14.3–16.5
Fantahun et al. [18]	16.4	15.2–17.6
Toru et al. [14]	16.8	15.7–18.0
Shewangizaw et al. [19]	17.5	16.4–18.7
Azale et al. [20]	25.4	23.3–27.6

The analysis is based on random effect model

### Statistical analysis of factors associated with postnatal depression

In the random effect model, we identified the following determinants or risk factors of PND. The odds of developing PND was higher among women who did not plan their pregnancy [AOR = 3.46, 95% CI (2.37–5.04)]. Women aged between 15 and 24 years were more likely to report symptoms of PND when compared to the older one [AOR = 1.72, 95% CI (1.11–2.68)]. Women who reported marital problems had an increased odds of PND [AOR = 3.07, 95% CI (2.36–3.99)]. Further, women experiencing the death of infant [AOR = 3.41, 95% CI (1.91–6.09)] and reported a history of substance use such as alcohol and Khat [AOR = 3.47, 95% CI (2.17–5.56)] were more likely to develop PND when compared to non-use. Moreover, the findings of this review also showed that getting low-income as explained by perceived financial hardship and experienced hunger in the past months significantly increased the odds of PND [AOR = 2.42, 95% CI (1.66–3.53)]. Poor social support, history of previous depression, domestic violence, and grand multiparty, perinatal complications, experiencing hunger in the preceding 1 month, stressful events, and history of abortion were factors significantly associated with postnatal depression in the respective studies not consistently.

### Publication bias

**Fig. 4:**
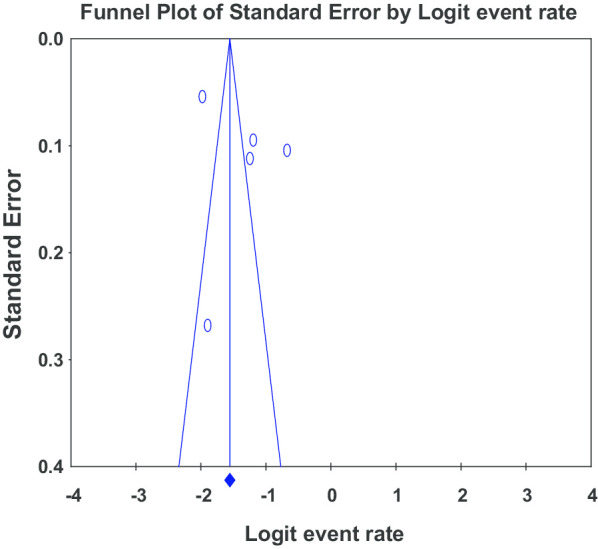
Funnel plot showing no publication bias of included studies

Publication bias may occurs in the systematic review and meta-analysis when the results of published studies are potentially different from the results of unpublished studies. The bias can represent all forms of bias such as outcome-reporting bias, gray-literature bias, time-lag bias, full-publication bias, citation bias, language bias as well as media-attention bias. This can be checked by the visual inspection of the funnel plot and Egger’s regression test. In this systematic review, both Egger’s regression test and visual inspection of the funnel plot, revealed no evidence of significant publication bias ((B = 6.98, SE = 6.51, P = 0.36) (Fig. 4).

## Discussion

This systematic review and meta-analysis included five studies with a total of 4751 study participants. The pooled estimated prevalence of postnatal depression in Ethiopia was 20.1%. Unplanned pregnancy, age between 15 and 24 years, marital problems, experiencing the death of the infant, history of substance use and having lower income were identified factors that were found to increase the odds of postnatal depression. Three studies were conducted in South Nation Nationalities and the People Regional States (SNNPRS), one from each was conducted in the Amhara region and Addis Ababa and produced inconsistent rates of prevalence. Therefore, the findings of this systematic review and meta-analysis are very important to guide postnatal clinical services and promote early screening and prevention of postnatal depression in Ethiopia.

The pooled prevalence estimate of this review was in line with studies conducted in South Africa [33, 34] and Nigeria [35]. However, the pooled estimated prevalence was higher than the studies' reports from Uganda [36] and Ghana [37] and lower than when compared to the reports from the study in South Africa [38]. The possible reasons for the observed difference in the prevalence of postnatal depression across the countries may be due to variations in socio-cultural and -economic factors, record keeping and reporting, and tools used to assess postnatal depression. For example, the studies conducted in South Africa and Uganda utilized Pitt depression inventory and Mini International Neuro-psychiatric Interview (MINI) respectively to assess postnatal depression. However, the studies included in our review used EPDS and PHQ9 only. Evidence from different studies also suggested that depressive symptoms in women increase in the first 2–12 weeks of delivery due to hormonal fluctuation and the new environment of childbirth [39, 40]. Thus, the interpretation of the estimates of this study should account for the window of measurement as a more precise window predicts more significant point estimates.

Unplanned pregnancy was associated with post-partum depression. In comparison to a planned pregnancy, women who did not plan their pregnancy were 3.46 times more likely to develop postnatal depression. This is in line with the studies conducted in Ghana and South Africa [41, 42]. An unplanned pregnancy may result from contraceptive failure and non-use of contraceptive services and, result in inadequate preparation for pregnancy and childbirth. This could exacerbate the physical health and psychological wellbeing of women and may result in postpartum depression. Further, women may develop postnatal depression due to stress which is associated with the transition into unplanned motherhood, suggesting strengthening the counseling services may contribute a significant role in alleviating a resultant depression. This is also complemented by a report from a prospective cohort study that suggested the minimal risk of depression was observed in women who planned their pregnancy [43].

In this review, women age between 15 and 24 years had 1.72 times the odds of having postnatal depression. The studies from Sudan also reported the same association [38]. This may be due to psychological distress as well as the stress of responsibility of marriage and childbirth. This is also supported by finding from a longitudinal study that reported the magnitude of depression was much higher among young people in their twenties [44].

We have also observed that women with marital problems such as divorced and widowed were prone to develop postnatal depression. Other studies also reported similar findings [44, 45]. However, the association between postnatal depression and divorce has not been explained yet. Nevertheless, some researchers hypothesized that depression by itself does not result in divorce and therefore, it is the result of not managing depression while others suggested that divorce alone can result in depression [46]. In contrast, a longitudinal study that used the data from the New Haven Epidemiologic Catchment Area (ECA) and investigated the association between marital problems and the risk of depressive episodes found a statistically significant association between marital disruption and the higher prevalence rates of depression in women [47].

The current review also found a high rate of postnatal depression among women experiencing the death of the infant. Findings from other studies in other countries also revealed a similar association. This may be due to losing an infant that can trigger intense feelings of grief and sadness. Having unmanaged feelings of grief and sadness after a loss could consequence postnatal depression. This is also supported by a cohort study that reported postnatal depression was significantly associated with approximately a three-fold increased risk of infant mortality [48].

Another important finding in the current review was that a remarkably higher rate of postnatal depression was observed among women with a history of a substance such as alcohol and khat use. A review of postpartum substance use and depression also supported this construct [49]. Different studies have been conducted so far revealed that women with postnatal depression are at a higher risk of developing substance abuse compared to women without postnatal depression.

### Limitations of the review

A small number of studies were included in the review and this might over-or under-estimate the pooled estimated prevalence of postnatal depression in Ethiopia. The review also lacked a sub-group analysis using a region as a moderator due to the small number of studies.

## Conclusion

The prevalence of postnatal depression in Ethiopia was high. Having unplanned pregnancy, age between 15 and 24 years, marital problems, experiencing death of infant, history of substance, poor social support, history of previous depression, domestic violence, grand multiparty, perinatal complications, experiencing hunger in the preceding 1 month, stressful events and history of abortion were factors significantly associated with postnatal depression. Therefore, the concerned body should give due attention to improve reproductive health services through early detection of risk factors of postnatal depression.

## Supplementary information

**Additional file 1:** Epidemiology of postnatal depression in Ethiopia: a systematic review and meta-analysis (Pre-designed protocol).

## Data Availability

All data generated or analyzed during this study are included in this article.

## Abbreviations

EPDS: Edinburgh Postnatal Depression Scale
LMICs: Low-income and middle-income countries
NOS: Newcastle Ottawa Scale
PHQ9: Patient Health Questionnaire
