# Supplementary material for: The epidemiology of postnatal depression in Ethiopia: a systematic review and meta-analysis
Source: Reprod Health. 2020 Nov 19;17:180. doi: 10.1186/s12978-020-01035-1 (PMC7678214; doi:10.1186/s12978-020-01035-1)
Supplement: Supplementary file 1 — Additional file 1: Epidemiology of postnatal depression in Ethiopia: a systematic review and meta-analysis (Pre-designed protocol). [file 12978_2020_1035_MOESM1_ESM.docx]

**Additional file 1: Epidemiology of postnatal depression in Ethiopia: a systematic review and meta-analysis (Pre-designed protocol)**

| **Review question** | What is the magnitude or prevalence of postnatal depression in Ethiopia?  What are the risk factors of postnatal depression? |
| --- | --- |
| **Types of study to be included** | This review will account all observational studies: cross sectional, case control, prospective and retrospective cohort studies |
| **Database searching** | Most commonly used databases such as PubMed, EMBASE, PSYCH INFO and GOOGLE Scholar will be used for searching the research question. In this study only observational study, with no publication date limit and English language studies will be included. |
| **Population** | All studies that reported the prevalence and/or associated factors of postnatal depression in Ethiopia will be included in the study. |
| **Exposure of interest** | Postnatal mothers who considered depressed as determined by any depression screening tool |
| **Outcome** | This review will include studies considered primary outcomes like; Depression during postnatal period measured using any valid and standard depression screening and rating scales. |
| **Data extraction** | We will search all the databases that are mentioned above and export all literature to endnotes. PRISMA statement will be used to guide and clearly present the study inclusion, exclusion, and reason for exclusion diagrammatically. |
| **Risk of bias (quality) assessment** | We will use Newcastle-Ottawa Scale (NOS) to assess the quality of studies that will be included in the Systematic review and meta-analysis. |
| **Data analysis** | The Comprehensive meta-analysis (CMA 3.0) software will be used to synthesize the data. |
|  | Subgroup and sensitivity analysis will also be conducted |
|  | Leave-one-out- sensitivity analysis also will be conducted if deemed. |
